# Usual intake of dairy products and the chance of pre-diabetes regression to normal glycemia or progression to type 2 diabetes: a 9-year follow-up

**DOI:** 10.1038/s41387-024-00257-7

**Published:** 2024-04-09

**Authors:** Zahra Bahadoran, Parvin Mirmiran, Fereidoun Azizi

**Affiliations:** 1 Nutrition and Endocrine Research Center, Research Institute for Endocrine Sciences, Shahid Beheshti University of Medical Sciences, Tehran, Iran; 2 Department of Clinical Nutrition and Dietetics, Faculty of Nutrition and Food Technology, National Nutrition and Food Technology Research Institute, Shahid Beheshti University of Medical Sciences, Tehran, Iran; 3 Endocrine Research Center, Research Institute for Endocrine Sciences, Shahid Beheshti University of Medical Sciences, Tehran, Iran

**Keywords:** Diabetes, Risk factors

## Abstract

**Background:**

We assessed the possible effect of usual dairy consumption on pre-diabetes (Pre-DM) remission or progression to type 2 diabetes (T2D).

**Methods:**

Pre-DM adults (*n* = 334, mean age of 49.4 years, and 51.5% men) were assessed for dairy intakes (2006–2008) and followed up to 9 years for incidence of T2D or normal glycemia (NG). All biochemical measurements were done at baseline and all subsequent examinations with 3-y follow-up intervals. Multinomial regression models with adjustment of confounding variables were used to estimate odds ratios (OR) and 95% confidence intervals (CIs) of incident T2D and NG for each serving/d dairy consumption.

**Results:**

The odds of NG was significantly elevated by 69% (OR = 1.69, 95% CI = 1.00–2.86, *P* = 0.05) per 200 g/d increased high-fat dairy intake, while the amount of total dairy or low-fat dairy was not related to the outcomes. Higher intakes of yogurt were more likely to be associated with an increased odds of NG (OR = 1.82, 95% CI = 1.20–2.74, *P* = 0.01). Usual intakes of milk, cheese, or cream-butter were not associated to Pre-DM remission or progression to T2D.

**Conclusion:**

Regular dairy consumption may increase the chance of Pre-DM regression to NG.

## Introduction

Pre-diabetes (Pre-DM), an intermediate hyperglycemic condition [1], is affecting about 27% of the middle-aged adults worldwide [2], and will surpass up to 470 million people by 2030 [3]. Distinct pathophysiologic pathways, i.e., obesity and insulin resistance or loss of β-cell function, are involved in occurrence of Pre-DM. Approximately, 5–10% of Pre-DM subjects progress to T2D annually [4, 5], corresponding to 70% within ten years [6]. Pre-DM subjects are highly at risk of developing cardiovascular diseases and premature death [7, 8].

Although T2D risk factors are well established, far less is known about potential moderators of Pre-DM regression and progression. Lifestyle modifications effectively prevent developing T2D in Pre-DM subjects by 36% (28–43%) [2]. Adherence to a healthy low-calorie, low-fat diet, and moderate physical activity level (PAL) results in Pre-DM remission by two-folds [9]. We recently reported that the chance of Pre-DM remission increased by 58% [OR = 1.58, 95% CI = 1.03–2.40] in subjects who had a PAL > 1500 MET-minutes/week [10], while having a Western-style dietary pattern significantly increased risk of developing T2D by 38% in Pre-DM subjects [11].

Regular intakes of dairy products, supplying protein and essential minerals (i.e., calcium, magnesium, potassium, and vitamin D) [12], have been reported can prevent developing Pre-DM and T2D [13, 14]. Whether dairy products may affect Pre-DM remission or progression to T2D is less documented [15]. Here, we aimed to investigate the association of usual intakes of dairy products and Pre-DM regression and progression in cohort of middle-aged adult participated in the Tehran Lipid and Glucose Study (TLGS). We also examined possible associations of different levels of dairy intake on long-term changes of glycemic parameters over 9 years.

## Material and methods

### Study population

This longitudinal study was conducted in an ongoing community-based prospective study (the Tehran Lipid and Glucose Study, TLGS), which started in 1999 on the 15,005 Tehranian residents, aged ≥3 years to investigate and prevent non-communicable diseases [16]. In this analysis, adult men and women (age ≥21 y) with Pre-DM (*n* = 334) who had completed data on usual diet, demographics, anthropometrics, and biochemical measurements in the third phase of the TLGS (2006-2008) were included, and followed up to a median of 9 years. Written informed consent was obtained from all participants. The ethics research council of the Research Institute for Endocrine Sciences, Shahid Beheshti University of Medical Sciences, Tehran, Iran, approved the study protocol (Ethics code: IR.SBMU.ENDOCRINE.REC.1401.080).

### Demographic, anthropometric, and biochemical measurements

Details of data collection and measurements of the variables (i.e., demographic, anthropometrics, blood pressure, medical history) in the TLGS have been reported elsewhere [16]. Details of biochemical measurements in the TLGS samples have been described elsewhere [17]. In brief, biochemical measurements [i.e., fasting serum glucose (FSG), triglyceride (TG), and high-density lipoprotein cholesterol (HDL-C] levels were all done after a 12-to 14-h overnight fast, at baseline and all subsequent examinations (with a 3-y follow-up intervals). The standard oral glucose tolerance test (OGTT) was performed for all adults (age≥21 y) who were not on glucose-lowering medications. Details of PAL measurements have been reported elsewhere [10].

### Dietary assessment

The usual dietary intakes of the participants over the previous year were assessed using a validated semi-quantitative 168-item food frequency questionnaire (FFQ) at baseline (2006–2008) [18]. Details of dietary assessment in the TLGS were described elsewhere [19, 20]. A residual adjustment was performed using a regression model (with total caloric intake as the independent variable and food/nutrients intake as the dependent variable) to mask the confounding effect of under-or over-report of energy intakes on estimated intakes of food groups and nutrients [21].

Dairy products were defined as stated by USDA and Food and Agriculture Organization (FAO) as “fluid beverage milk, fermented milk, cheese, butter and cream, ice cream, yogurt, dry milk products, condensed milk, and whey products”. Low-fat dairy intakes (g/d) was calculated as the sum of low-fat milk and low-fat yogurt, and high-fat dairy was calculated as the sum of high-fat milk, chocolate milk, high-fat yogurt, cream yogurt, regular and cream cheese, cream and butter. Total dairy consumption (g/d) was calculated as the sum of low- and high-fat dairy products. One serving of each dairy products was converted as follows: 120 g for milk and yogurt; 28 g for cream cheese and regular cheese; 5 g for cream and butter; and 120 g for ice cream.

### Definition of terms

Pre-DM was defined as having at least one of the IFG (100 ≤ FSG < 126 mg/dL) or IGT (140 ≤ 2h-SG < 200 mg/dL) [1]. NG was defined as the first occurrence of both normal fasting glucose and normal glucose tolerance (NFG, i.e., FSG < 100 and NGT, i.e., 2h-SG < 140); T2D was defined as the first occurrence of FSG ≥ 126 mg/dL or 2h-SG ≥ 200 mg/dL, or using glucose-lowering medications. A positive family history of T2D was defined as having at least one parent or sibling with T2D. Details of calculating T2D-risk score (a strong predictor of T2D in our population [22]), and its efficacy to improve stability of the multivariate models have reported elsewhere [23].

### Statistical methods

Statistical analyses were conducted using the SPSS for Windows version 20 (SPSS Inc., Chicago, IL, USA). Baseline characteristics of the participants were illustrated across the groups using analysis of variance (ANOVA) or Kruskal-Wallis test. Repeated-measures generalized estimating equation (GEE) linear regression models were used to estimate the overall mean of FSG and 2h-SG overtime across.

The odds ratios (95% confidence intervals, CIs) of Pre-DM regression to normal glycemia or progression to T2D in relation to usual intakes of dairy products were estimated using multinomial logistic regression analysis. Potential covariates were selected based on both statistical and scientific evidence. A univariate analysis was performed for potential confounding variables, and those with *P*_E_ < 0.2 were selected for the final multivariable model; *P*_E_ (*P*-value for entry) determines which variables should be included in the multivariable model. Finally, three logistic models, including crude model, adjusted-model 1 (adjusted for subjects’ age, time-to-event, sex, 2h-SG, and T2D-risk score), and adjusted-model 2 (additionally-adjusted for intakes of fruits, vegetables, legumes-nuts, grains, meats, smoking habits, and physical activity) were conducted.

## Result

The mean age of the study participants was 49.4 ± 12.8 y, and 51.5% were men. The rate of Pre-DM progression and regression was similar (39.8%) during a median follow-up of 8.9 years (inter-quartile range: 7.0–9.6 years). The baseline characteristics of the study participants are summarized in Table 1. Compared to those progressed to T2D, participants who returned to normal glycemia were younger and had lower serum glucose levels, BMI and TG-to-HDL-C ratio. Table 2 summarizes the baseline daily dietary intakes of the participants. No significant difference was observed between the groups; compared to those who remained at Pre-DM state, daily intakes of high-fat dairy products were more likely to be higher (mean = 208 ± 239 *vs*. 150 ± 161 g/d) in subjects who returned to normal glycemia. Higher median daily intakes of cheese (serving/d) were observed in subjects who developed T2D during the follow-up period.

**Table 1:** Baseline characteristics of the study participants according to outcome (*n* = 334).

	Normal glycemia (*n* = 133)	Pre-DM (*n* = 68)	T2D (*n* = 133)
Age *(y)*	45.2 ± 14.0^a,b^	51.5 ± 11.7	51.3 ± 11.2
Men *(%)*	48.9	55.9	51.9
FH *(%)*	23.5	17.5	26.8
Medications			
Lipid-lowering *(%)*	0.6	2.9	5.3
BP-lowering *(%)*	5.3	5.9	9
Current smoker *(%)*	14.3	14.9	13.7
Physical activity *(MET-h/week)*	26.0 ± 29.1	21.4 ± 23.6	23.6 ± 24.2
Low-physical activity^c^ *(%)*	42.1	42.2	39.7
BMI *(kg/m*^*2*^*)*	28.9 ± 4.6	28.9 ± 4.5	29.5 ± 4.8
WC *(cm)*	96.3 ± 11.3	98.1 ± 10.1	100 ± 10.3
SBP *(mm Hg)*	119 ± 16.2	121 ± 16.8	124 ± 18.1
DBP *(mm Hg)*	77.0 ± 9.5	78.5 ± 11.8	79.6 ± 11.8
FSG *(mg/dL)*	98.2 ± 9.7^b^	101 ± 8.4^b^	105 ± 8.9
2h-SG *(mg/dL)*	125 ± 30.0^b^	131 ± 35.6^b^	145 ± 31.3
TG to HDL-C ratio	4.3 ± 3.2^b^	5.2 ± 3.6	5.4 ± 3.8

Data are mean ± SD (unless stated otherwise).

^a^Significant difference with Pre-DM (*P* < 0.05).

^b^Significant difference with T2D (*P* < 0.05) (analysis of variance (ANOVA) was used).

^c^Physical activity<600 *MET-min/week*.

*T2D* type 2 diabetes, *FH* Family history of T2D, *BMI* body mass index, *WC* waist circumference, *SBP* systolic blood pressure, *DBP* diastolic blood pressure, *FSG* fasting serum glucose, *2h-SG* 2-hours serum glucose, *TG* serum triglyceride, *HDL-C* high-density lipoprotein cholesterol, *NFG-NGT* Normal glycemia, normal fasting glucose-normal glucose tolerance.

**Table 2:** Dietary intakes of the study participants according to the outcome status (*n* = 334).

	Normal glycemia (*n* = 133)	Pre-DM (*n* = 68)	T2D (*n* = 133)
Total energy intakes *(kcal/d)*	2357 ± 877	2215 ± 886	2344 ± 899
Total fats *(g/d)*	82.6 ± 39.4	80.9 ± 47.3	81.6 ± 44.2
Protein *(g/d)*	83.4 ± 51.4	78.5 ± 40.0	84.5 ± 43.8
Carbohydrates *(g/d)*	342 ± 139	319 ± 139	355 ± 203
Calcium *(mg/d)*	1305 ± 568	1243 ± 636	1247 ± 568
Phosphorous *(m2357g/d)*	1574 ± 838	1449 ± 768	1554 ± 783
Total dairy products *(g/d)*	473 ± 318	439 ± 331	445 ± 324
Low-fat dairy products *(g/d)*	262 ± 198	289 ± 264	273 ± 250
High-fat dairy products *(g/d)*	208 ± 239^a^	150 ± 161	159 ± 189
Milk *(serving/d)*	0.64 (0.27–1.92)	0.68 (0.27–1.92)	0.42 (0.06–1.92)
Yogurt *(serving/d)*	1.73 (0.82–2.20)	1.10 (0.54–1.93)	1.45 (0.56–1.97)
Cheese *(serving/d)*	0.71 (0.26–1.00)	0.54 (0.28–1.00)	0.85 (0.28–1.26)^b^
Cream-butter *(serving/d)*	0.43 (0.17–1.70)	0.64 (0.07–1.57)	0.38 (0.07–1.10)

Data are mean ± SD or median (inter-quartile ranges, IQR).

Servings of milk, yogurt, chees, and cream-butter were defined as 120 mL, 120 mL, 30 g and 5 g, respectively.

^a^Significant difference with Pre-DM group (*P* < 0.05), analysis of variance (ANOVA) was used.

^b^Significant difference with Pre-DM group (P < 0.05), Kruskal-Wallis test was used.

*T2D* type 2 diabetes, *FH* Family history of T2D, *BMI* body mass index, *WC* waist circumference, *SBP* systolic blood pressure, *DBP* diastolic blood pressure, *FSG* fasting serum glucose, 2h-SG 2-hours serum glucose, *TG* serum triglyceride, *HDL-C* high-density lipoprotein cholesterol, *NFG-NGT*, Normal glycemia, normal fasting glucose-normal glucose tolerance.

Table 3 shows the mean ± SD of glycemic parameters (FSG and 2h-SG concentrations) across the low, medium, and high-level intakes of dairy products (defined as tertile categories), during the study follow-up. Participants with higher intakes of high-fat dairy products (≥230 *vs*. < 50 g/d) had a borderline significant lower 2h-SG concentrations over time (150, 95% CI = 141–159 *vs*. 165, 95% CI = 148–166 mg/dL, *P* = 0.063); no significant difference was observed between the tertile categories of the total, low-fat and high-fat dairy products for FSG over the time. Similar analyses conducted for different types of dairy products showed that higher intakes of cheese (≥ 1 *vs*. < 0.4 serving/d) were associated with higher 2h-SG concentrations over time (164, 95% CI = 151–177 *vs.*149, 95% CI = 145–156 *vs*.  mg/dL, *P* = 0.061); compared to its first tertile (<0.2 serving/d), higher intakes of milk (median of 0.5 and 1.9 serving/d) were related to lower 2h-SG concentrations over time (149 and 155 mg/dL *vs*. 170, *P* = 0.005 and *P* = 0.066, in the second and third tertiles compared to the first tertile). No significant difference was observed between levels of different dairy products with FSG over time.

**Table 3:** Mean FSG and 2h-SG concentrations (mg/dL) across tertiles of daily intakes of dairy products over 9 years of follow-up.

	Baseline (2006–2008)	First follow-up (2009–2011)	Second follow-up (2012–2014)	Third follow-up (2015–2017)	Overall mean (95% CI)	*P*_time×group_
**Total dairy products** ***(g/d)***						
<295 (median = 180)						
FSG	101 ± 9.2^a^	110 ± 18.6	108 ± 18.9	116 ± 55.0	111 (107–114)	0.658
2h-SG	138 ± 35.5^b^	152 ± 55.0	169 ± 69.6	195 ± 85.0	163 (154–172)	0.295
295-540 (median = 390)						
FSG	101 ± 9.7	108 ± 18.9	112 ± 26.4	114 ± 31.4	109 (105–112)	
2h-SG	135 ± 30.5	146 ± 54.1	162 ± 63.6	180 ± 74.2	156 (147–165)	
≥ 540 (median = 700)						
FSG	110 ± 9.7	108 ± 22.3	113 ± 23.0	113 ± 25.2	109 (106–112)	
2h-SG	129 ± 32.4	141 ± 50.3	164 ± 65.4	179 ± 64.8	153 (144–162)	
**Low-fat dairy products** ***(g/d)***						
<140 (median = 70)						
FSG	101 ± 9.9	109 ± 20.5	112 ± 25.7	115 ± 35.5	109 (106–112)	0.940
2h-SG	136 ± 35.2	148 ± 57.7	162 ± 66.5	186 ± 81.3	158 (149–167)	0.691
140–300 (median = 250)						
FSG	101 ± 9.1	110 ± 22.4	113 ± 26.8	113 ± 31.6	109 (106–112)	
2h-SG	137 ± 30.5	150 ± 52.2	169 ± 68.2	185 ± 75.3	160 (151–169)	
≥300 (median=470)						
FSG	102 ± 9.6	108 ± 16.7	114 ± 23.1	115 ± 28.6	110 (107–113)	
2h-SG	130 ± 32.9	141 ± 49.4	164 ± 63.8	183 ± 69.4	154 (146–164)	
**High-fat dairy products** ***(g/d)***						
<50 (median = 25)						
FSG	102 ± 9.0	110 ± 18.8	114 ± 24.9	116 ± 29.8	110 (107–114)	0.602
2h-SG	139 ± 34.6	155 ± 60.7	171 ± 67.6	196 ± 76.7	165 (156–174)	0.070
150-230 (median=110)						
FSG	101 ± 9.9	110 ± 17.0	114 ± 23.9	114 ± 35.2	109 (106–113)	
2h-SG	135 ± 33.1	146 ± 45.7	166 ± 61.9	183 ± 73.4	157 (148–166)	
≥230 (median=290)						
FSG	101 ± 9.7	107 ± 23.6	111 ± 26.9	113 ± 30.6	108 (105–111)	
2h-SG	129 ± 30.5	138 ± 51.1	158 ± 68.7	176 ± 75.0	150 (141–159)^*^	

Data are mean ± SD. The generalized estimating equation (GEE) was used.

^a^Mean ± SD of FSG.

^b^Mean ± SD of 2h-SG ^*^P (for overall mean difference between tertile 3 *vs*. tertile 1) = 0.06.

The odds ratio (95% CI) of Pre-DM regression and progression in relation to dairy intakes are presented in Table 4. The chance of returning to normal glycemia was significantly increased by 69% (OR = 1.69, 95% CI = 1.00–2.86, *P* = 0.05) per 200 g/d high high-fat dairy intake, independent of the well-established risk factors of T2D development. The total or low-fat dairy amount was not related to the chance Pre-DM regression to a normal state or progression to T2D. Higher intakes of yogurt were more likely to be associated with an increased chance of returning to normal glycemia (OR = 1.82, 95% CI = 1.20–2.74, *P* = 0.01). Higher intakes of cheese were more likely to be associated with a 2-folds elevated risk of developing T2D among Pre-DM subjects (OR = 2.08, 95% CI = 1.01–4.30, *P* = 0.04) in the second model, however, after adjustment of dietary factors the significant association was disappeared. Daily intakes of milk and cream-butter were not related to the chance of regression/progression of Pre-DM.

**Table 4:** The odds ratio (95% CI and *P* value) of Pre-DM regression to normal glycemia and progression to T2D in relation to intakes of dairy products.

	Normal glycemia	T2D
Total dairy		
*Crude*	1.06 (0.88–1.28, *P* = 0.49)	1.01 (0.84–1.22, *P* = 0.89)
*Model 1*	1.02 (0.83–1.23, *P* = 0.85)	1.05 (0.86–1.28, *P* = 0.61)
*Model 2*	1.05 (0.78–1.41, *P* = 0.40)	1.09 (0.82–1.44, *P* = 0.57)
*Model 3*	1.13 (0.84–1.54, *P* = 0.41)	1.07 (0.79–1.44, *P* = 0.66)
Low-fat dairy		
*Crude*	0.91 (0.71–1.15, *P* = 0.43)	0.95 (0.75–1.20, *P* = 0.65)
*Model 1*	0.89 (0.68–1.15, *P* = 0.37)	0.98 (0.75–1.25, *P* = 0.88)
*Model 2*	0.86 (0.66–1.12, *P* = 0.53)	0.99 (0.76–1.27, *P* = 0.94)
*Model 3*	0.99 (0.67–1.46, *P* = 0.97)	0.94 (0.64–1.38, *P* = 0.76)
High-fat dairy		
*Crude*	1.34 (0.96–1.86, *P* = 0.08)	1.07 (0.75–1.51, *P* = 0.69)
*Model 1*	1.22 (0.86–1.72, *P* = 0.25)	1.11 (0.78–1.60, *P* = 0.53)
*Model 2*	1.65 (1.00–2.72, *P* = 0.05)	1.28 (0.77–2.12, *P* = 0.32)
*Model 3*	1.69 (1.00–2.86, *P* = 0.05)	1.28 (0.75–2.17, *P* = 0.35)
Milk		
*Crude*	1.04 (0.59–1.80, *P* = 0.89)	0.69 (0.40–1.18, *P* = 0.18)
*Model 1*	0.94 (0.53–1.67, *P* = 0.85)	0.75 (0.43–1.31, *P* = 0.32)
*Model 2*	1.38 (0.66–2.87, *P* = 0.38)	1.08 (0.55–2.13, *P* = 0.83)
*Model 3*	1.34 (0.62–2.94, *P* = 0.45)	0.80 (0.38–1.66, *P* = 0.55)
Yogurt		
*Crude*	2.08 (0.76–5.65, *P* = 0.15)	1.66 (0.62–4.48, *P* = 0.31)
*Model 1*	1.98 (0.70–5.55, *P* = 0.19)	1.62 (0.53–4.07, *P* = 0.35)
*Model 2*	1.44 (0.47–4.37, *P* = 0.52)	1.13 (0.37–3.48, *P* = 0.82)
*Model 3*	1.82 (1.20–2.74, *P* = 0.01)	1.33 (0.89–1.99, *P* = 0.15)
Cheese		
*Crude*	1.14 (0.65–2.02, *P* = 0.63)	1.49 (0.82–2.71, *P* = 0.18)
*Model 1*	1.16 (0.65–2.09, *P* = 0.60)	1.55 (0.58–4.51, *P* = 0.35)
*Model 2*	1.79 (0.85–3.75, *P* = 0.24)	2.08 (1.01–4.30, *P* = 0.04)
*Model 3*	1.35 (0.63–2.81, *P* = 0.43)	1.54 (0.72–3.24, *P* = 0.25)
Cream-butter		
*Crude*	1.03 (0.63–1.68, *P* = 90)	0.86 (0.52–1.39, *P* = 53)
*Model 1*	1.01 (0.60–1.69, *P* = 0.93)	0.89 (0.53–1.49, *P* = 0.62)
*Model 2*	1.34 (0.66–2.71, *P* = 0.41)	0.94 (0.47–1.88, *P* = 0.87)
*Model 3*	1.10 (0.83–1.46, P = 0.48)	0.96 (0.67–1.23, P = 0.57)

Data are ORs and 95% CI (Multinomial logistic regression was used).

Model 1, adjusted for age, sex, 2h-SG, and T2D-risk score; Model 2, additionally adjusted for smoking and physical activity; Model 3, additionally adjusted for dietary intakes of fruits, vegetables, legumes-nuts, grains, and meats.

For total dairy, low-fat and high-fat dairy, ORs (95% CIs) correspond to each 200 g/d. The logarithm of serving for dairy products (i.e., milk, yogurt, cheese, cream-butter) was included in the model (due to non-normal distribution of the serving values).

Pre-DM, pre diabetes; T2D, type 2 diabetes mellitus; Normal glycemia, normal fasting glucose-normal glucose tolerance.

Daily intakes of milk, yogurt, or cream-butter were not related to regression or progression from Pre-DM.

## Discussion

We observed a significant association between regular high-fat dairy consumption and the chance of regression to normal glycemia in a 9-year follow-up of Pre-DM subjects. Each 200 g/d of high-fat dairy corresponded to an elevated chance of returning to normal glycemia by 69%; higher intakes of high-fat dairy were also related to a lower 9-years average of 2h-SG among Pre-DM adults. A median consumption of 0.5 and 1.9 serving of milk per day, compared to its daily intake of <0.2 servings, was related to a better post-prandial glycemia over time. We further noted a significant positive association between daily consumption of yogurt and the chance of Pre-DM regression.

The causality and underlying mechanisms of the observed relations between dairy intake and the risk of developing T2D in cohort studies remain unclear [24]. Both protective and neutral effects are documented [15, 25–29]. Regular dairy consumption in relation to the risk of Pre-DM progression to T2D has been less established, and no evidence is available connecting dairy products to the chance of regression from Pre-DM to normal glycemia. High-fat dairy showed evidence of a dose-response and an inverse association with incident T2D in a 12-year follow-up of Pre-DM subjects (70% reduced risk, in relation to ≥14 *vs*. < 1 serving/week [25]. In a recent global observational study among 21 countries (low, middle, and high-income nations), the protective effect of whole-fat compared with low-fat dairy against the risk of T2D was numerically more strong [26]. Likewise, the Framingham Heart Study Of spring Cohort reported that higher high-fat dairy consumption decreased the risk of Pre-DM progression by 70% [25]. A part of the protective effects of dairy products in relation to the risk of T2D is attributed to their fatty acids profiles (medium-chain, odd, very long-chain SFAs, and *trans*-palmitoleic acid) [30]; however, other bioactive components, including probiotics, menoquinones, and milk fat globule membrane have also received a significant attention [24]. Biomarkers of dairy fat consumption, including C15:0, C17:0, and transC16:1n7, were significantly related to reduced risk of T2D (0.80, 95% CI = 0.73–0.87; 0.65, 95% CI = 0.59–0.72; 0.82 95% CI = 0.70–0.96) [31]. A dose-response meta-analysis of seventeen cohort studies, however, reported a neutral association between high-fat dairy and developing T2D (0.98, 95% CI = 0.94, 1.03) per 200 g high-fat dairy products/d) [28].

We did not find a significant association between low-fat and total fat dairy consumption and the chance of Pre-DM regression or progression. A recent report from the TLGS research group also indicated that a 3-years change in dairy intakes might modify the risk of developing T2D among Pre-DM adults. A decreased consumption of total dairy (> 0.5 servings/day) compared with a remaining stable state was related to an elevated chance of Pre-DM progression (OR = 1.56, 95%CI = 1.02–2.41), while increasing low-fat dairy consumption (especially milk and yogurt) by 0.50 serving/d was associated with a lower risk of T2D (OR = 0.56, 95% CI = 0.35–0.90) [15]. A pooled relative risk of 0.93 (95% CI = 0.87, 0.99) per 400 g total dairy products/d and 0.91 (95% CI = 0.86, 0.96) per 200 g low-fat dairy products/d was reported with a non-linear trend flattening of the curve at higher intakes [28].

Similar controversies are observed in observational studies connecting different dairy products with the risk of T2D. Non-linear inverse associations were found for daily consumption of yogurt (80 *vs*. 0 g/d, RR = 0.86, 95% C = 0.83, 0.90) and ice cream (for each 10 g/d, RR = 0.81, 95% CI = 0.78, 0.85) [32]; these inverse associations were not linear and no incremental benefits were found at a higher intake [32]. Pooled estimated relative risks of seventeen cohorts indicated a protective effect for cheese (RR = 0.92, 95% CI = 0.86–0.99, per 50 g cheese/d), and neutral effects for either milk (RR = 0.87, 95% CI = 0.72–1.04, per 200 g milk/d) and yogurt (RR = 0.78, 95% CI = 0.60–1.02, per 200 g yogurt/d) [28]. An updated meta-analysis also reported that higher yogurt consumption was significantly associated with decreased risk of T2D (OR = 0.83, 95% CI = 0.73–0.94) [29]. A 12-year follow-up of Pre-DM subjects showed that cheese consumption has a dose-response and an inverse association with incident T2D (63% reduced risk, in relation to ≥4 *vs*. < 1 serving/week for cheese) [25], while a neutral association between cheese intake and risk of T2D (RR = 1.00 per 10 g/d, 95% CI = 0.99–1.02) was obtained by a more recent meta-analysis [32]. A possible adverse effect of high cheese intake on glucose metabolism is supported by evidence indicating that dietary patterns with a high load of cheese consumption increase the risk of gestational diabetes, obesity, and abdominal obesity [33, 34]. In our study, the amount of yogurt consumption was meaningfully higher from a dietitian’s point of view in subjects who returned to normal glycemia compared to those who remained Pre-DM (1.73 *vs*. 1.1 serving/d), and higher intake of yogurt was related to reverting Pre-DM to normal glycemia.

The inconsistent findings for cheese and yogurt in the literature have been attributed to their complex and heterogeneous nature, a large variety of dairy products in different countries, and to differences between how these foods are eaten in diverse populations (e.g., hard cheeses consumed with fruit and nuts, plain whole-fat yogurt in the Spanish people, versus the use of processed cheese on pizza and deli meat sandwiches, or sugar-sweetened low-fat yogurt in US population). Our FFQ did not differentiate various types of cheeses (e.g., Lighvan, Koozeh, Gouda, Cumin, Feta, Cheddar, Iranian white cheese) and they were just categorized as regular and cream cheese, while previous reports attributed the different metabolic effects to multiple kinds of cheeses (e.g., fermented *vs*. non-fermented cheese, Dutch *vs*. curd cheese) [35]. Furthermore, we believed that the high-sodium content of cheese (~377–600 mg of sodium per serving, considered a significant contributor to total daily sodium intake [36, 37]) might explain some adverse effect of cheese, since high-sodium intake is a risk factor for developing T2D [38, 39]. Possible contamination of cheese with histamine-producing bacteria during cheese processing and storage [40], may also be a potential risk for developing T2D [41].

Due to the different underlying pathophysiology of IGT and IFG, the effects of diet on pre-DM progression and regression may differ for these subgroups. IFG is coincidental with reduced hepatic insulin sensitivity, β-cell dysfunction and mass reduction, altered glucagon-like peptide-1 secretion, and elevated glucagon secretion. In contrast, IGT is accompanied by a reduced peripheral insulin sensitivity with almost normal hepatic insulin sensitivity, a progressive loss of β-cell function, decreased secretion of the glucose-dependent insulinotropic polypeptide, and elevated glucagon secretion [7, 42]. Because of the relatively low sample size and lack of enough power, we could not differentiate between the potential effects of dairy intakes on the chance of regression/progression of Pre-DM in the isolated-IGT, isolated-IFG, or combined IFG-IGT subgroups. Because of distinct etiologies of isolated-IFG and isolated-IGT (relevance to genetic factors, smoking, and male sex, *vs*. physical inactivity, unhealthy diet, and short stature [42]), one can speculate that our findings would be more related to IGT rather than IFG state. Strong correlations between 2h-SG may support this idea compared to FSG with dairy intakes, especially high-fat dairy products over time.

Some strengths and limitations should be considered to interpret the study findings. Data collection use of a valid and reliable semi-quantitative 168-FFQ reduced the possibility of reporting biases. The well-known risk factors of T2D were detected and controlled in our analyses, however, due to existing of other possible unknown risk factors, complete controlling for confounders was not possible in our models. Repeated measurements of glycemic parameters and other covariates during the study follow-up, at the 3-year intervals, enabled us to monitor the study participants’ glycemic changes more precisely over time and detect the occurrence of the outcomes at mid-interval periods.

To sum up, our findings in the TLGS cohort provide some evidence against previous claims on adverse effects of whole-fat dairy products on cardiometabolic risk factors and support the last reported protective effect of high-fat dairy against the development of T2D, as increased high-fat dairy consumption over the course of follow-up were related to a considerably increased chance of Pre-DM regression to normal glycemia. Inversely, higher consumption of cheese was associated with the risk of developing T2D. These findings further support that regular consumption of dairy may attenuate the risk of developing T2D or the chance of returning to normal glycemia, and various dairy products may affect these pathways differently. Furthermore, our findings may indicate that the effects of regular dairy intake on the risk of developing T2D or the chance of returning to normal glycemia in Pre-DM subjects are mediated through improving glucose tolerance and insulin sensitivity, as dairy intake was associated with repeated measures and overall mean of 2h-SG rather FSG, over time.

## Data Availability

Data will be presented upon forwarding the request to the corresponding author (mirmiran@endocrine.ac.ir) and confirmation of the director of RIES (azizi@endocrine.ac.ir).
